# A case of breast cancer developed in the chronic expanding hematoma cyst wall

**DOI:** 10.1016/j.radcr.2024.07.184

**Published:** 2024-08-23

**Authors:** Ayami Sudo, Shoji Oura

**Affiliations:** Department of Surgery, Kishiwada Tokushukai Hospital, Kishiwada-city, OSAKA, 596-8522, Japan

**Keywords:** Breast cancer, Case report, Chronic expanding hematoma, Hemosiderin, Hemosiderin laden macrophage

## Abstract

No studies have reported breast cancer cases developed in the chronic expanding hematoma (CEH). Case presentation: A 47-year-old woman was referred to our hospital for the treatment of a large breast mass. Ultrasound showed that the tumor had an intra-cystic tumor pattern. Magnetic resonance imaging (MRI) of the mass component showed a hypo intense pattern on T1-weighted images, a mosaic pattern on T2 weighted images, and a faint enhancement on time-signal intensity images. Core needle biopsy pathologically showed connective tissue, hematoma, and hemosiderin laden macrophages neither with any mammary gland components nor with malignant cells. These image findings and the presence of hemosiderin laden macrophages led us to the diagnosis of CEH despite the lack of prior breast surgery. The large tumor size of the presumed CEH and its tendency for rapid growth made us attempt to treat the breast lesion with lumpectomy. Frozen section, however, revealed malignant cells in the hard part of the capsule, leading to the conversion of breast surgery from lumpectomy to nipple-preserving mastectomy. Postoperative pathological study showed that the tumor was composed of atypical cells growing in cribriform, tubular, and papillary fashions. In addition to the malignant cells, cyst wall of the CEH had abundant fibrosis and hemosiderin deposits. This is the first CEH case after no prior breast surgery, which had breast cancer within it. When a breast CEH is suspected, careful follow-up is imperative to avoid underestimating a possible concomitant breast cancer.

## Introduction

Chronic expanding hematomas of the breast (CEHs) are rare disorders generally observed after some kind of breast operation such as breast cancer surgery or breast augmentation [[1], [2], [3]]. No studies, however, have reported CEHs without prior breast surgery to date.

Mammography and ultrasound are very useful in the diagnosis of breast cancer. However, when the background gland is dense breast, mammography may not be able to detect even relatively large breast cancers. Ultrasound can show tomographic plane and depict even small breast cancers in the dense breast, but often fails to detect them due to the impossible visualization of the whole breast at once. In contrast, except for minute ductal carcinoma in situ, magnetic resonance imaging (MRI) can show more small breast cancers without micro calcifications than mammography and ultrasound [4].

We herein report a T2 breast cancer case, unable to have been detected even with MRI, having existed in the CEH cyst wall in a patient with no history of breast surgery.

## Case report

**Fig. 1:**
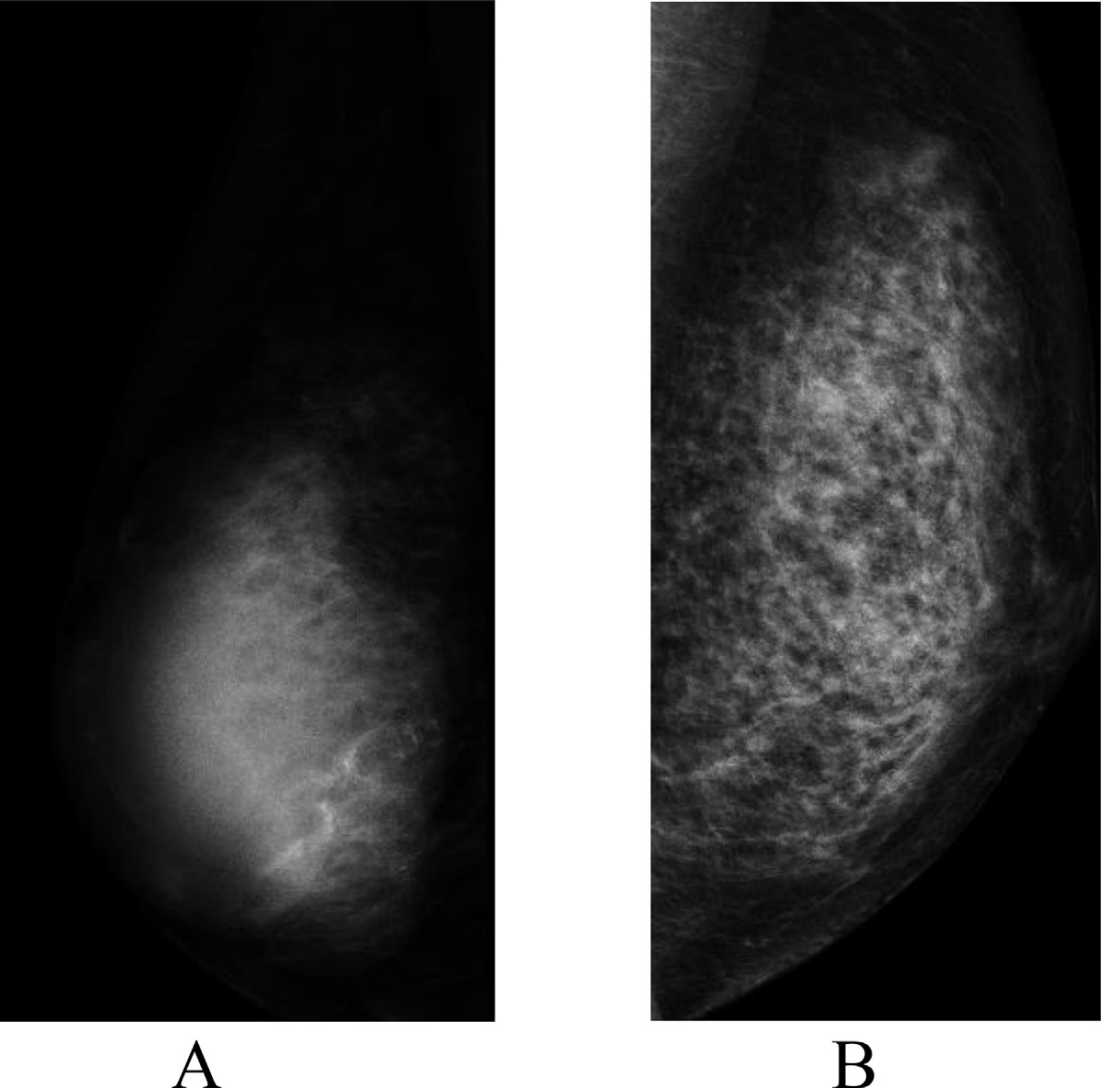
Mammography findings. Mammography showed global asymmetry between the right (A) and left (B) breasts.

**Fig. 2:**
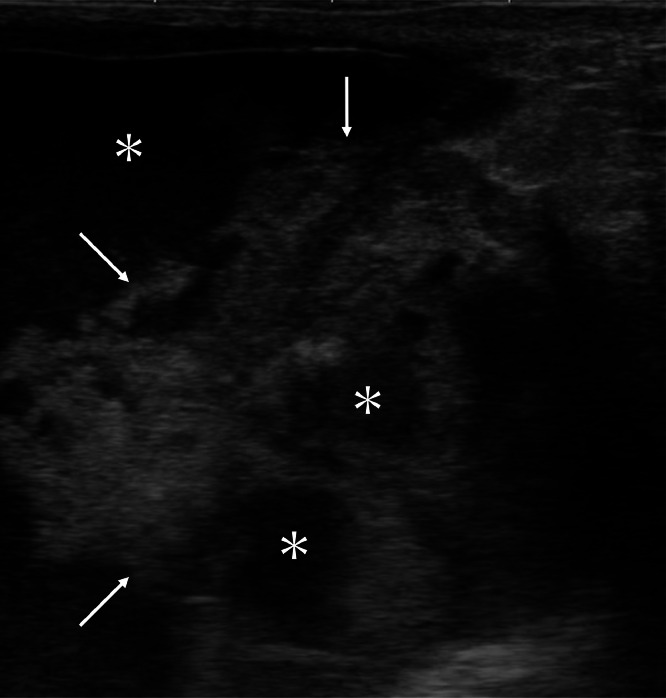
Ultrasound findings. Ultrasound showed hyper echoic mass components (arrows) and cystic parts (asterisks).

**Fig. 3:**
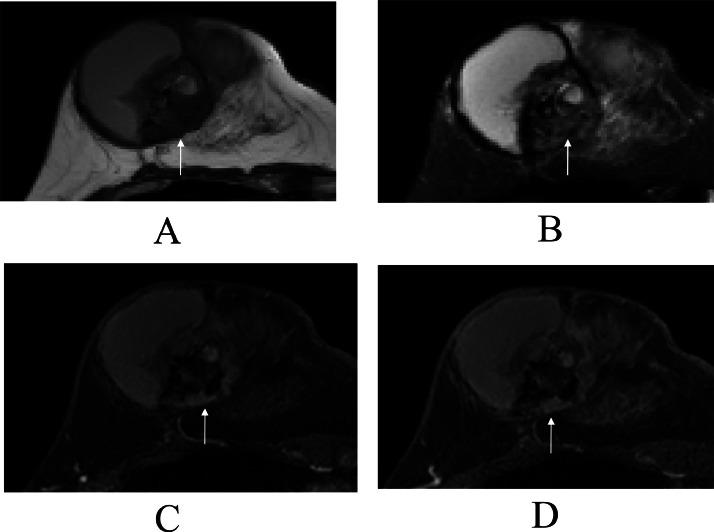
Magnetic resonance imaging (MRI) findings. MRI of the mass components (arrow) showed a hypo intense pattern on T1-weighted images (A) and a mosaic pattern on T2-weighted images (B). Enhanced MRI of the mass components (arrow) showed a faint enhancement pattern both on early (C) and late (D) phase images.

**Fig. 4:**
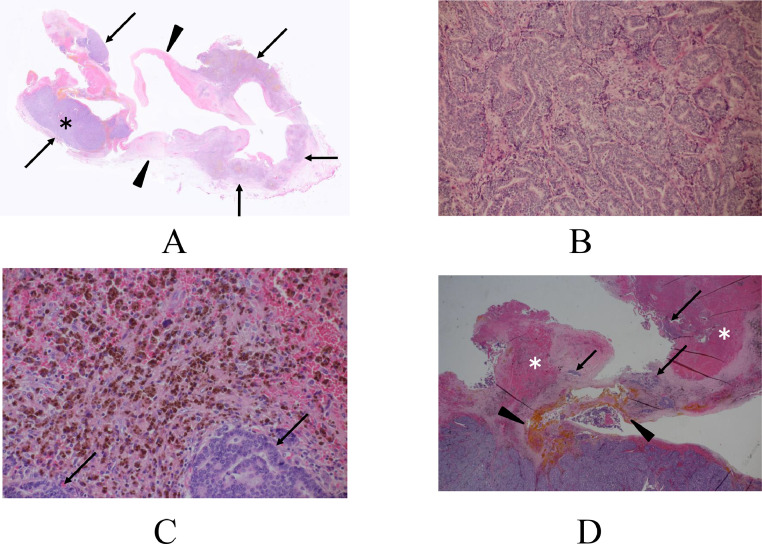
Pathologic findings. (A) Low magnified view showed a ruptured fibrous capsule with (arrows) and without (arrowheads) scattered micro cancer nodules. (B) Magnified view showed atypical cells growing mainly in a tubule forming fashion. (C) Magnified view showed that numerous hemosiderin laden macrophages (brownish cells) encompassed the cancer cell nests (arrows). (D) Low magnified view showed scattering cancer cell nests (arrows) and hematomas (asterisks). Numerous hemosiderin deposits were observed around the cancer cell nests (arrowheads).

A 47-year-old Japanese unmarried woman without any cancer family history had noticed a breast mass for more than 2 years. However, rapid enlargement of the mass made the patient visit a hospital. Mammography showed global asymmetry of her right mammary gland (Fig. 1). Ultrasound showed that the mass had an intra-cystic tumor pattern (Fig. 2). Magnetic resonance imaging (MRI) of the mass component showed a hypo intense pattern on T1-weighted images, a mosaic pattern on T2 weighted images, and a faint enhancement on time-signal intensity images (Fig. 3). Cytological study of the intra-cystic fluid showed no malignant cells. Pathological study of the intra-cystic mass component showed connective tissue and hematoma with various components such as micro vessels, lymphocytes, neutrophils, and hemosiderin laden macrophages, but neither malignant cells nor normal breast ducts / lobules. These image and pathologic findings led us to the diagnosis of CEH despite the absence of prior breast surgery. The large tumor size of the presumed CEH and its tendency for rapid growth made us attempt to treat the breast lesion with lumpectomy. The patient, therefore, was scheduled to undergo a lumpectomy followed by immediate breast reconstruction using latisimus dorsi musculocutaneous (LDMC) flap based on the patient's strong preference to avoid breast deformity. Frozen section, however, revealed malignant cells in the hard part of the capsule, leading to the conversion of breast surgery from lumpectomy to nipple-preserving mastectomy. LDMC flap reconstruction was also converted to extended LDMC reconstruction [5] for better cosmetic outcome. Additive sentinel node biopsy showed no lymph node metastasis. Postoperative pathological study showed that the tumor was composed of atypical cells growing in cribriform, tubular, and papillary fashions (Fig. 4). In addition to the malignant cells, cystic walls of the CEH had abundant fibrosis and hemosiderin deposits. Immunostaining showed that the tumor was luminal type invasive ductal breast cancer. The patient has been well on tamoxifen therapy with favorable cosmetic outcome for 11 months.

## Discussion

Mammography showed only a large mass image and did not further contribute to the preoperative diagnosis in this case. Ultrasound, however, clearly depicted the intra-cystic tumor pattern. Ultrasound of the mass component showed a high echo pattern, generally observed in the intra-cystic breast tumors [6,7]. This finding should have been caused by massive back scattering of ultrasound waves due to the markedly different acoustic impedance of the liquid component and the connective tissue in the CEH.

In the diagnosis of intra-cystic breast tumors, diagnostic physicians always pay much attention how the mass component arises from the cyst wall to judge whether the solid part is benign or malignant. However, based on the postoperative pathological findings, malignant cells were present even in the areas that were judged to be simple cyst walls. The large mass component had attracted our attention, possibly having led us to the insufficient evaluation of the cyst walls. When diagnosing large intra-cystic breast lesions using ultrasound, it is necessary for physicians to observe the entire lesion including the presumed benign cystic parts in detail.

Almost all studies of the breast CEH have reported the presence of prior breast surgery. This patient, however, had not received any breast operations. In addition, the patient had no bleeding tendency, was unmarried and without a partner, and had no history of breast trauma, even minor ones. Therefore, why CEH occurred in this case remained uncertain.

Invasive breast cancer component was larger than 2cm in this case and was composed of cancer cells without any interminglement of fibrous, cartilage, and bone components in the cancer cell nests. Cell-rich tumors generally show a hyper intense pattern on T2-weighted images and a washout pattern on time-signal intensity images, both of which were not observed in this case. Hemosiderin belongs to paramagnetic substances and had pathologically surrounded the cancer cell nests in this case. Hemosiderin has overwhelmingly higher magnetic susceptibility than other many paramagnetic substances, and is also called a super-paramagnetic substance. Therefore, the massive presence of hemosiderin laden macrophages just around the breast cancer cell nests caused the hypo intense pattern on T2-weighted images and brought about no meaningful tumor enhancement. Harada et al. also reported only faint tumor enhancement on MRI in a large breast cancer, 60mm in size, with marked hemosiderin deposits before chemotherapy. The case further showed a large susceptibility artifact, even precluding the interpretation of MRI images, in and around the regressed tumor after neoadjuvant chemotherapy [8].

Ko et al. [9] and Cotesta et al. [10] reported post-traumatic recurrent hemorrhagic cysts in breast cancer patients. The former study did not provide any detailed pathological findings, but the latter study showed that the entire cyst wall was formed by grade 3 cancer cells. In this case, the hemorrhagic cyst wall was formed, at least to some extent, by a noncancerous fibrous capsule. It, therefore, is reasonable to conclude that in this case, breast cancer existed in the cyst wall of the CEH.

## Conclusions

This is the first breast cancer case having existed in the CEH cyst wall in a patient with no prior breast surgery. When a breast CEH is suspected, careful follow-up is imperative to avoid underestimating a possible concomitant breast cancer.

## Author contributions

AS designed the concept of this study. SO drafted the manuscript.

## Patient consent

Written informed consent was obtained from the patient for the publication of this case report and any accompanying images.
